# Detection of Gene Fusions in Soft Tissue Sarcoma Using Next-Generation Sequencing

**DOI:** 10.3390/genes17050514

**Published:** 2026-04-27

**Authors:** Piotr Remiszewski, Klaudia Bobak, Jakub Piątkowski, Paweł Golik, Andrzej Tysarowski, Katarzyna Seliga, Mateusz J. Spałek, Anna Szumera-Ciećkiewicz, Michał Wągrodzki, Piotr Rutkowski, Anna M. Czarnecka

**Affiliations:** 1 Department of Soft Tissue/Bone Sarcoma and Melanoma, Maria Sklodowska-Curie National Research Institute of Oncology, 00-001 Warsaw, Poland; piotrremiszewski789@gmail.com (P.R.); klaudia.bobak@pib-nio.pl (K.B.); mateusz.spalek@pib-nio.pl (M.J.S.);; 2 Medical Faculty, Medical University of Warsaw, 02-091 Warsaw, Poland; 3 Faculty of Biology, Institute of Genetics and Biotechnology, University of Warsaw, 02-091 Warsaw, Poland; 4 Cancer Molecular and Genetic Diagnostics Laboratory, Maria Sklodowska-Curie National Research Institute of Oncology, 00-001 Warsaw, Poland; 5 Genetics and Genomics Centre, National Medical Institute of the Ministry of the Interior and Administration, 02-507 Warsaw, Poland; 6 Department of Radiotherapy I, Maria Sklodowska-Curie National Research Institute of Oncology, 00-001 Warsaw, Poland; 7 Department of Pathology, Maria Sklodowska-Curie National Research Institute of Oncology, 00-001 Warsaw, Poland; 8 Biobank, Maria Sklodowska-Curie National Research Institute of Oncology, 00-001 Warsaw, Poland; 9 Department of Experimental Pharmacology, Mossakowski Medical Research Institute, Polish Academy of Sciences, 02-093 Warsaw, Poland; 10 Outpatient Chemotherapy Department, Maria Sklodowska-Curie National Research Institute of Oncology, 00-001 Warsaw, Poland

**Keywords:** sarcoma genetics, molecular targets, gene fusions, real-world, chemotherapy response, therapeutic targets

## Abstract

Introduction: Soft tissue sarcomas (STS) exhibit profound molecular heterogeneity. While recurrent gene fusions hold significant diagnostic and therapeutic value—guiding treatment selection and identifying novel molecular targets—our understanding of their broader clinical implications remains limited. Materials and Methods: We performed next-generation sequencing (NGS; FusionPlex Sarcoma v2, Archer™) and bioinformatic analysis (STAR v.2.7, Arriba) on formalin-fixed paraffin-embedded (FFPE) core needle biopsy specimens. The cohort consisted of patients enrolled in a phase II clinical trial (NCT03651375) who received preoperative chemoradiotherapy according to the UNRESARC protocol. Results: The analysed cohort comprised nine adult patients (median age 66 years; range 44–73) diagnosed with undifferentiated pleomorphic sarcoma (UPS; *n* = 3), malignant peripheral nerve sheath tumour (MPNST; *n* = 3), myxofibrosarcoma (MFS; *n* = 2), and leiomyosarcoma (LMS; *n* = 1), predominantly high-grade (G3; 5/9) and extremity-localised (6/9). Gene fusions were detected in one-third of patients (3/9), exclusively in G3 tumours. Specifically, we identified an *SGSH-PRKCA* fusion in MFS (thigh), a *LINC01133-OGA* fusion in MPNST (thorax), and a concurrent *JAZF1-MYH7B* (chr7:27995037 intronic-chr20:33563203 exon/splice-site, out-of-frame but preserving myosin domains) with a PRKCA-associated intergenic rearrangement (chr1, retaining C1/kinase domains) in UPS (upper back). Notably, the *SGSH-PRKCA* and *JAZF1-MYH7B* pairs have not been previously described in the literature for these STS subtypes. Fusion-positive (F1) cases showed stable radiological disease (RECIST 1.1 SD) and EORTC C/D pathological responses with 5–20% residual viable tumour, whereas fusion-negative (F0) cases showed a wider range of radiological and pathological outcomes, including partial response, progression, and stable disease. Conclusions: Our analysis suggests that broad genomic profiling may provide complementary molecular information in diagnostically challenging cases managed at specialised sarcoma centres, particularly when morphology and immunohistochemistry are insufficient. In the present series, however, the detected rearrangements did not alter systemic treatment, and the data do not support claims of prognostic, predictive, or therapeutic actionability.

## Simple Summary

Soft tissue sarcoma (STS) is characterised by profound molecular heterogeneity and encompasses over 100 recognised histological subtypes, with a five-year overall survival (OS) of approximately 16–25% in advanced disease. Canonical gene fusions, present in 33–50% of sarcomas, provide diagnostic and, in selected subtypes, prognostic information; however, their predictive value with respect to conventional cytotoxic chemotherapy remains unestablished. Conventional diagnostic techniques such as FISH and RT-PCR are restricted to a predefined panel of target genes and are labour-intensive, whereas RNA-based next-generation sequencing (NGS) enables simultaneous detection of known and novel rearrangements from limited FFPE material. In this nine-patient adult cohort treated with standard neoadjuvant doxorubicin–ifosfamide within the UNRESARC phase II trial (NCT03651375), targeted mRNA sequencing identified gene fusions in 33.3% (3/9) of cases, all confined to G3 tumours; these findings did not alter the treatment delivered. The detected rearrangements—SGSH-PRKCA, LINC01133-OGA, and concurrent JAZF1-MYH7B/PRKCA-associated rearrangements—are of unknown functional and clinical significance and should be regarded as exploratory. Larger, prospectively annotated cohorts with orthogonal validation are required before any prognostic or predictive inference can be drawn.

## 1. Introduction

Sarcomas are malignancies of mesenchymal origin, arising from osteocytes, adipocytes, chondrocytes, muscle cells, fibroblasts, neural cells, and stromal cells [1,2]. This cellular diversity is known for producing profound histological and molecular heterogeneity, with over 100 recognised subtypes [3]. Advanced STS is characterised by a five-year OS of 16–25%, and standard systemic therapy relies primarily on doxorubicin and the alkylating agent ifosfamide [4,5,6]. Although targeted alternatives remain scarce, these conventional cytotoxic agents continue to form the cornerstone of neoadjuvant and metastatic management due to their established, albeit limited, objective response rates. Anthracycline–ifosfamide has remained in clinical use not because it is biologically selective, but because, despite modest efficacy, it has historically provided reproducible activity across multiple high-risk STS settings in the absence of consistently superior systemic alternatives [4,5,6,7]. Clinical trials face substantial challenges from STS heterogeneity and rarity; consequently, evidence of therapeutic efficacy is often limited to real-world cohorts rather than large randomised controlled trials [7,8].

Across sarcomas, driver gene fusions (GFs), present in between 33% and 50%, are known to guide histopathological subclassification, refine prognosis, and, in selected contexts, predict response to targeted therapies. GFs and copy number alterations (CNVs) act as primary oncogenic drivers, especially in translocation-associated subtypes, while broad CNVs characterise complex-karyotype entities such as undifferentiated pleomorphic sarcoma (UPS) and malignant peripheral nerve sheath tumour (MPNST). The utilisation of fusion testing has been instrumental in establishing diagnoses in cases that are otherwise challenging to diagnose, for instance, in extraskeletal myxoid chondrosarcoma—now widely recognised as a distinct *NR4A3-*rearranged soft-tissue sarcoma, rather than chondrosarcoma subtype [9,10,11,12,13,14,15].

The rarity of sarcoma complicates the evaluation of drug efficacy in clinical trials for the various histological subtypes associated with respective GFs. Many fusion genes are enriched in particular histotypes and can be diagnostically informative, although a given fusion is not necessarily exclusive to a single subtype. Consequently, experiments utilising patient-derived cell lines or xenografts carrying the fusion gene may be influenced by factors external to the fusion gene itself [16].

Conventional methods (e.g., FISH, RT-PCR) are limited in terms of targeted genes and tissue-intensive in contrast to underused next-generation sequencing (NGS) panels [17]. Moreover, NGS allows for the discovery of novel fusions as no pre-specified targets are needed [18,19]. Therefore, we present a small retrospective case series from a national sarcoma reference centre to describe mRNA-detected gene rearrangements in adult STS treated with standard neoadjuvant anthracycline–ifosfamide within the UNRESARC trial. The aim was exploratory and descriptive: to assess whether targeted RNA sequencing could reveal additional molecular findings in this setting, rather than to demonstrate prognostic, predictive, or immediate therapeutic utility.

## 2. Materials and Methods

All histopathological diagnoses were established according to the contemporaneous World Health Organisation (WHO) Classification of Soft Tissue and Bone Tumours by specialist sarcoma pathologists, based on FFPE core needle biopsy specimens; no open surgical biopsies were included. We performed targeted mRNA sequencing using the FusionPlex Sarcoma v2 assay (Archer™) on a NextSeq500 platform (Illumina, San Diego, CA, USA). The bioinformatic pipeline comprised the following steps: (i) adapter trimming and quality filtering of raw reads; (ii) alignment to the hg38 human reference genome using STAR (v2.7); (iii) fusion detection and annotation using Arriba (v2.4), which applies blacklisting of recurrent artefacts, discordant read-pair filtering, and confidence scoring; (iv) manual review of all candidate fusions for chromosomal breakpoint coordinates, reading-frame prediction, retained functional domains, and coverage. The technology utilised is RNA-based anchored multiplex PCR (AMP) with unidirectional gene-specific primers and unique molecular identifier barcodes, covering 63 sarcoma-relevant genes including known fusion partners, canonical and alternative splice sites, hotspot exons, and key coding regions. This design enables simultaneous detection of known and novel GFs, splice variants, single-nucleotide variants (SNVs), small insertions/deletions, and relative transcript expression. No orthogonal validation—including fluorescence in situ hybridisation (FISH) or RT-PCR—was performed for detected fusions, which constitutes a key limitation. RNA yield, library concentration, and sequencing depth followed manufacturer-specified thresholds; however, individual run-level quality metrics are not reported here. Because the present analysis was restricted to targeted mRNA sequencing, broader genomic features such as co-occurring somatic mutations, mutational burden, and copy-number alterations were not systematically analysed and are therefore outside the scope of the current report.

The specimens were obtained from patients treated in a clinical trial (NCT03651375) according to the UNRESARC protocol, which included neoadjuvant chemotherapy and radiotherapy followed by resection [20]. Adults with marginally resectable, intermediate- or high-grade soft-tissue sarcoma of the extremities or trunk wall, ECOG 0–2 and adequate organ function were included in the study. Following the amendment of the study protocol, patients with 1–5 resectable lung metastases were also deemed eligible to participate. Key exclusions included lymph-node involvement or unresectable metastases, prior overlapping radiotherapy, second active malignancy, contraindications to anthracycline-ifosfamide (AI) or radiotherapy, and specific histologies (Ewing sarcoma, rhabdomyosarcoma, osteosarcoma, GIST, DFSP, angiosarcoma, aggressive fibromatosis). The treatment plan encompassed three 21-day AI cycles, comprising doxorubicin at a dosage of 25 mg/m^2^ on days 1–3 and ifosfamide at a dosage of 2.5 g/m^2^ on days 1–4, administered concurrently with mesna and pegfilgrastim. Subsequent to the completion of cycle 1, patients underwent hypofractionated radiotherapy at a dose of 25 Gy in 5 fractions, administered within a period of 1 week. Following this, a limb-sparing or conservative surgical intervention was performed, whenever feasible, 6–8 weeks later. Postoperative boosts of 30–40 Gy were permitted for R1 margins [20].

Crucially, the RNA-based NGS results from the present analysis were not available to the treating clinicians during the neoadjuvant phase; accordingly, the treatment plan was not modified on the basis of fusion status in any patient. Clinical data analysis was descriptive owing to the small sample size. Continuous variables are presented as medians with ranges, and categorical variables as counts and proportions. No inferential statistical testing was performed.

## 3. Results

This adult STS cohort (*n* = 9) is characterised by predominantly high-grade disease arising mainly in the extremities, with gene fusions detected in one-third of cases and radiological responses largely limited to a small subset of fusion-negative, intermediate-grade tumours. Median age at diagnosis was 66 years (range 44–73), with a slight male predominance (5/9; 55.6%). Tumour sites were predominantly located in the limbs (6/9), including the forearm (*n* = 2), arm (*n* = 2), thigh (*n* = 2), and leg (*n* = 1), with the remaining cases involving axial locations (upper back *n* = 1, thorax *n* = 1) and one deep-seated thigh leiomyosarcoma.

**Figure 1:**
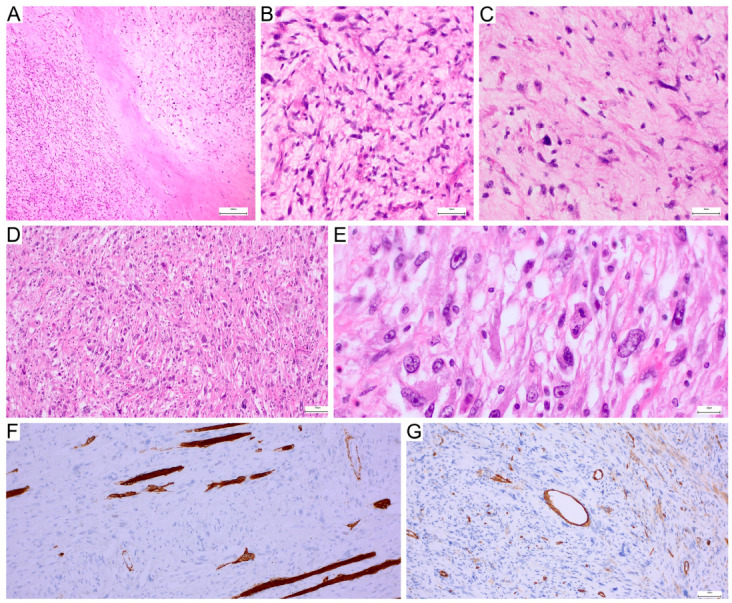
Histopathological features of high-grade MFS and UPS. (**A**–**C**) High-grade MFS showing infiltrative growth in a myxoid stroma (**A**). Panel (**B**) demonstrates a more cellular area composed of pleomorphic spindle to stellate tumour cells with nuclear atypia, whereas panel (**C**) shows a more prominently myxoid and less cellular area. (**D**,**E**) UPS composed of highly atypical pleomorphic spindle and epithelioid tumour cells arranged in a storiform to haphazard pattern (**D**), with marked nuclear pleomorphism seen at higher magnification (**E**). (**F**) Desmin immunohistochemistry in undifferentiated pleomorphic sarcoma, with strong positivity restricted to residual skeletal muscle fibres and no expression in tumour cells. (**G**) CD34 immunohistochemistry highlighting vascular endothelial cells, with only very weak reactivity in a subset of neoplastic cells.

The histological spectrum comprised UPS (*n* = 3), MPNST (*n* = 3), MFS (*n* = 2), and LMS (*n* = 1). Grading showed a predominance of high-grade tumours: grade 3 (G3) in 5/9 (55.6%; UPS *n* = 2, MPNST *n* = 2, MFS *n* = 1), grade 2 (G2) in 3/9 (33.3%; one each of MPNST, MFS, UPS), and one ungraded LMS (11.1%). G2 tumours were confined to extremity or limb-girdle sites (forearm MPNST, arm MFS, arm UPS), whereas G3 lesions involved both extremity (forearm UPS, leg MPNST, thigh MFS) and axial locations (upper back UPS, thoracic MPNST). This distribution is consistent with a clinically advanced cohort in which both anatomical accessibility and biological aggressiveness vary across subtypes (Figure 1).

Targeted RNA-sequencing identified fusion-positive (F1) status in 3/9 patients (33.3%), all of whom had G3 tumours, and fusion-negative (F0) status in the remaining 6/9 (66.7%). The F1 group encompassed three distinct histologies and sites: a G3 UPS of the upper back, a G3 MPNST of the thorax, and a G3 MFS of the thigh. Detected rearrangements included: a *JAZF1-MYH7B/PRKCA*-associated fusion in UPS, in which genomic mapping localised the JAZF1 breakpoint to an intronic region at chr7:27995037 and the *MYH7B* breakpoint to an exon/splice-site at chr20:33563203. This transcript is characterised by an out-of-frame configuration or premature stop-codon, with domain-level annotation suggesting partial preservation of myosin-related regions. However, the out-of-frame structure and lack of protein-level validation preclude conclusions regarding translation into a functional chimeric protein. The concurrent PRKCA-associated rearrangement mapped to an intergenic locus on chromosome 1, retaining the phorbol-ester/diacylglycerol-binding C1 domain and the kinase C-terminal domain.

In addition, a LINC01133-OGA fusion was identified in G3 MPNST, and an SGSH-PRKCA fusion was detected in G3 MFS, with domain-level annotation suggesting retention of selected sulfatase- and kinase-related regions. Call-level support varied across the reported rearrangements: SGSH-PRKCA showed medium-confidence support with 14 split reads and local coverage of 113 and 2461 reads at the respective breakpoints, whereas LINC01133-OGA showed medium-confidence support with two split reads and coverage of 0 and 541 reads. The UPS case harboured multiple JAZF1-MYH7B transcript candidates with heterogeneous support, including a high-confidence call at chr7:27995037-chr20:33563203 supported by 1 and 1 split reads and a medium-confidence call at chr7:28031528-chr20:33563203 supported by 0 and 42 split reads. However, the absence of frame confirmation, the heterogeneous support across candidate transcript calls, and the lack of protein-level validation preclude conclusions regarding functional chimeric protein formation.

These complex structural variants illustrate the molecular heterogeneity of high-grade STS and show how RNA-based profiling may contribute to the molecular characterisation of morphologically pleomorphic entities, although its diagnostic impact depends on validation and clinical context. Importantly, structural annotation based on RNA sequencing alone does not establish functional protein expression. In the present series, several rearrangements were predicted to be out-of-frame, and therefore any inferred domain preservation should be interpreted cautiously, as translation into functional chimeric proteins remains unproven.

### 3.1. Radiological Response

Radiological response was assessed by Response Evaluation Criteria in Solid Tumours (RECIST) 1.1 [21]. Objective responses were uncommon; the objective response rate (ORR) was 22.2% (2/9), comprising solely partial responses (PR) in two G2, fusion-negative, extremity sarcomas—a forearm MPNST and an arm MFS—both intermediate-grade (G2) and F0. Stable disease (SD) was the predominant radiological outcome, recorded in 66.7% (6/9) of cases and encompassing both F0 and F1 tumours across multiple histologies (UPS, MPNST, MFS) and anatomical sites. Progressive disease (PD) was confined to a single ungraded, fusion-negative thigh LMS (11.1%). Overall, 88.9% (8/9) of patients achieved disease control (PR or SD), suggesting that the neoadjuvant regimen was characterised predominantly by tumour stabilisation; radiologically measurable shrinkage was limited to a small subset of intermediate-grade extremity lesions.

### 3.2. Pathological Response

**Figure 2:**
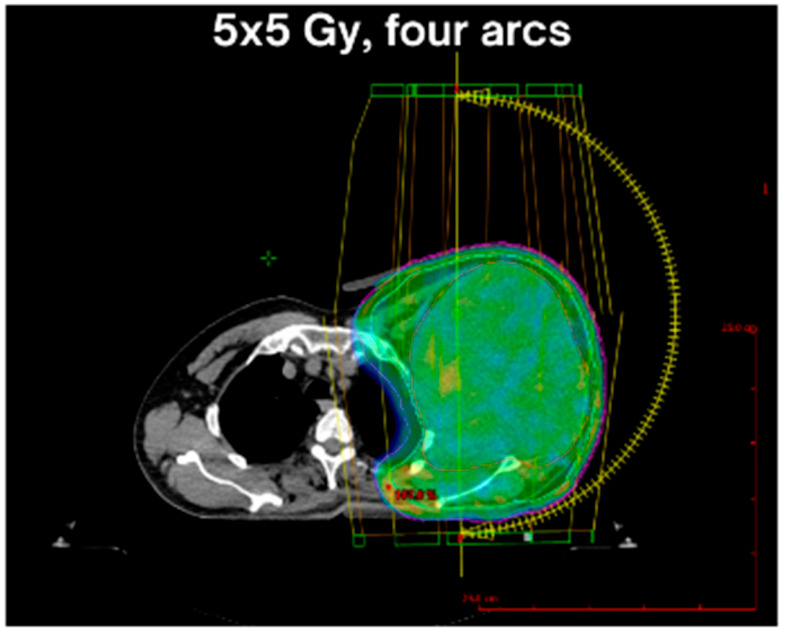
Volumetric modulated arc therapy (VMAT) treatment plan for a patient with G2 UPS of the shoulder (P5). VMAT plan illustrates dose distribution of hypofractionated RT (5 × 5 Gy) within the UNRESARC protocol [20].

**Figure 3:**
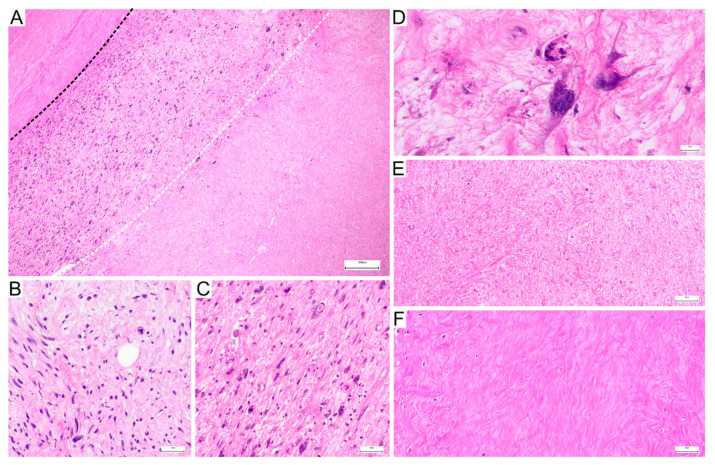
Histopathological features of malignant peripheral nerve sheath tumour (MPNST) before and after treatment. (**A**) Post-treatment specimen showing three distinct zones: hyalinizing fibrosis above the black dashed line, residual viable tumour between the dashed lines, and necrosis below the white dashed line. The viable residual tumour is identified by scattered atypical spindle cells with elongated hyperchromatic nuclei, in contrast to the paucicellular hyalinized fibrotic area and the acellular eosinophilic necrotic zone. (**B**) Pretreatment MPNST with a cellular spindle cell proliferation composed of elongated, wavy, hyperchromatic nuclei. (**C**) Post-treatment MPNST with prominent therapy-related changes and residual neoplastic spindle cells remains evident. (**D**) High-power view of the treated lesion shows residual atypical tumour cells within a myxoid-to-collagenized background. (**E**) Overview of treated tumour tissue with extensive necrosis. (**F**) Dense hyalinized fibrosis consistent with treatment effect.

Pathological response was evaluated using European Organisation for Research and Treatment of Cancer (EORTC) criteria [22], with quantitative assessment of residual viable tumour (RVT). No complete pathological responses were observed. EORTC grade E (no effect; RVT 70–90%) was recorded in 33.3% (3/9) of patients, grade D (minor effect; RVT 10–35%) in 44.4% (4/9), and grade C (moderate effect; RVT 5–9%) in 22.2% (2/9). Grade E cases comprised a G3 fusion-negative UPS of the forearm (RVT 90%), a G3 fusion-negative MPNST of the leg (RVT 70%), and a G2 fusion-negative MFS of the arm (RVT 70%). Grade D cases included the ungraded F0 thigh LMS (RVT 30%), a G2 F0 arm UPS (RVT 35%) (Figure 2), a G3 F1 upper-back UPS harbouring *JAZF1-MYH7B*/*PRKCA*-associated rearrangement (RVT 20%), and a G3 F1 thigh MFS harbouring *SGSH-PRKCA* (RVT 10%). Grade C was observed in the G2 F0 forearm MPNST (RVT 9%) and the G3 F1 thoracic MPNST with *LINC01133-OGA* (RVT 5%). When molecular, radiological, and pathological readouts are considered together, fusion-positive G3 tumours consistently achieved SD radiologically and showed low-to-intermediate RVT (5–20%; EORTC C/D), whereas the two PRs arose exclusively in fusion-negative G2 extremity sarcomas with divergent histological clearance. Fusion-negative G3 tumours, particularly UPS and MPNST, showed predominantly none-to-minor pathological effects with high RVT (up to 90%) (Figure 3). These descriptive observations illustrate the complex, non-linear relationship between molecular status, grade, anatomical site, imaging-based response, and microscopic tumour clearance in high-grade STS; no causal interpretation can be drawn from this sample size (Table 1).

## 4. Discussion

Despite extensive immunohistochemistry, gene fusion analysis remains essential in rare/challenging STS diagnoses [23,24]. Key fusions in sarcoma comprise SS18::SSX (synovial sarcoma), FUS::DDIT3 (myxoid liposarcoma), COL1A1::PDGFB (dermatofibrosarcoma protuberans), PAX3/7::FOXO1 (alveolar rhabdomyosarcoma), NAB2::STAT6 (solitary fibrous tumour), EWSR1::FLI1 (Ewing sarcoma) [1,13,23,25,26,27,28,29]. Despite extensive immunohistochemistry, gene fusion analysis remains important in diagnostically challenging STS. In this setting, RNA-based NGS can identify both canonical and atypical rearrangements that may refine classification, although the clinical significance of newly detected fusions in complex-karyotype sarcomas remains uncertain without orthogonal confirmation and functional validation. For example, Bobak et al. identified novel *PRUNE2::NTRK2* fusions (two out-of-frame isoforms/case; exon16/8–exon20 breakpoints) in two adult STS (LMS/MFS) via NGS (TruSight Oncology 500/FusionPlex Sarcoma v2); both frameshifted—yielding non-functional *TrkB* (STOP codon kinase domain) with unclear P*RUNE2* (tumour suppressor) integrity [30]. Despite advanced disease, both achieved >7-year survival post-standard neoadjuvant chemoradiotherapy and multiple lines of chemotherapy. *NTRK* fusions typically drive oncogenesis/targetable by larotrectinib/entrectinib—yet this atypical variant’s suppressor vs. dominant-negative impact remains undefined [30]. In one retrospective analysis of 7494 patient samples across 44 histologies, targeted panel sequencing refined 10.5% of initial diagnoses [18]. Potentially actionable genetic alterations were observed in 31.7% (*n* = 2372) of patients. In particular, kinase gene rearrangements were identified in 2.6% (*n* = 196) of cases, and a tumour mutational burden (TMB) of ≥10 mut/Mb was present in 3.9%. The genomic landscape is known for low microsatellite instability (<0.3%, *n* = 18) but high genome-wide loss of heterozygosity (gLOH) in 15% (*n* = 697) of evaluable sarcomas, which is not fully explained by the 2.5% (*n* = 184) homologous recombination deficiency rate. This first real-world MENA sarcoma cohort (*n* = 78) confirms NGS feasibility, identifying targetable alterations in 42% and NBTA-associated PFS benefit (9 vs. 2 months; *p* = 0.023), aligning with global series showing 10–30% actionable rates and HR 0.4–0.7 for matched therapy [11]. Late NGS timing (73%) and access barriers underscore the need for upfront profiling, particularly in chondrosarcoma with CDKN2A/B deletions. Other series report similar findings: for example, a Chinese cohort (*n* = 145) using a 558-gene panel found gene fusions in 40% of cases [31], while a multicentre Turkish series (*n* = 81) detected therapeutically actionable alterations in 22.2% and revised the histopathologic diagnosis in 4.9% [14]. Further institutional data demonstrated that targeted NGS identified pathogenic alterations in 60% of a 20-case series, resulting in diagnostic and therapeutic changes in 15% (*n* = 3/20). Identification of fusions such as ETV6-NTRK3 and FUS-TFCP2 validates the capacity of NGS to resolve diagnostic ambiguity and direct clinical management [32]. Overall, broad profiling identifies targets (kinase fusions, mutations, biomarkers) in a substantial minority of STS, and has a measurable impact on diagnosis and treatment planning.

Notably, as per the 2024 ESMO Precision Medicine Working Group recommendations, exploratory NGS for novel alterations or phase I/II trial enrolment remains confined to specialised reference centres [26,33]. However, routine care has evolved: in non-specialised institutions, broad genomic profiling is now strongly justified when the primary aim is to resolve ambiguous histopathological diagnoses in complex soft tissue sarcomas, or screen for established ESCAT Level I/II targets (e.g., ALK fusions in inflammatory myofibroblastic tumour; COL1A1::PDGFB in DFSP) and tumour-agnostic markers (MSI-H/dMMR, TMB-H, NTRK fusions) in the advanced metastatic setting (Table 2) [26,33].

We present a real-world, single-centre series of nine adult STS patients treated with standard neoadjuvant AI within the UNRESARC phase II trial, in whom targeted mRNA sequencing was applied retrospectively [20]. Because AI is a conventional cytotoxic agent rather than a molecularly targeted therapy, and because the NGS results were not used to guide treatment in any patient, no definitive correlation between fusion status and response to chemotherapy can be established from this dataset. The detected fusions—SGSH-PRKCA, LINC01133-OGA, and concurrent JAZF1-MYH7B/PRKCA-associated rearrangements—are not previously described for the respective STS subtypes in the published literature and therefore remain of unknown functional and clinical significance (Table 3). In complex-karyotype entities such as UPS and MFS, the genomic background is dominated by pervasive CNAs rather than subtype-defining fusions; the present data suggest that RNA profiling may occasionally reveal structural rearrangements even in these tumours, but out-of-frame configurations, absence of protein-level validation, and low coverage at the PRKCA-intergenic breakpoint preclude any inference of functional activity [34,35]. Moreover, experimental evidence indicates that kinase fusions involving PKC family members are paradoxically loss-of-function in some oncological contexts rather than constitutively activating, further undermining a straightforward actionability interpretation. These findings should therefore be framed as descriptive and hypothesis-generating rather than as evidence of clinical utility.

Several limitations constrain the interpretation of these findings. The cohort is small (*n* = 9), derived from a single reference centre, and spans four histological subtypes and two grades, which precludes consistent cross-subtype comparisons. Fusion calls were based exclusively on targeted mRNA sequencing without orthogonal confirmation by FISH or RT-PCR; out-of-frame predictions and low-coverage calls carry a material false-positive risk. The broader genomic context—co-occurring somatic mutations and CNVs—was not assessed in the present dataset, limiting mechanistic interpretation. Survival endpoints including progression-free survival (PFS) and OS were not evaluable, so the long-term prognostic implications of the observed radiological and pathological responses remain undefined. Finally, and most importantly, the NGS data were not used to inform treatment decisions; the series therefore provides no evidence that fusion testing modifies clinical management or patient outcomes in this specific setting. Accordingly, all findings must be interpreted as descriptive and hypothesis-generating, and the series does not support claims of immediate clinical utility.

## 5. Conclusions

STS is characterised by marked molecular heterogeneity, with complex-karyotype subtypes such as UPS, MPNST, and MFS presenting particular diagnostic and therapeutic challenges. In this nine-patient adult series, targeted mRNA sequencing identified gene rearrangements in 33.3% (3/9) of cases, all confined to G3 tumours: SGSH-PRKCA in MFS, LINC01133-OGA in MPNST, and concurrent JAZF1-MYH7B/PRKCA-associated rearrangements in UPS. These fusion pairs have not been previously described for these specific histotypes. However, the small sample size, absence of orthogonal validation, out-of-frame configurations of several rearrangements, and exclusive use of conventional non-targeted neoadjuvant chemotherapy preclude any prognostic, predictive, or therapeutic interpretation. The NGS findings did not alter clinical management in any patient. RNA-based profiling may provide complementary diagnostic information in selected morphologically ambiguous STS managed at specialist reference centres; however, in this cohort, it did not modify treatment, and no prognostic, predictive, or therapeutic inference should be drawn without orthogonal validation in larger, prospectively annotated, histotype-stratified series.

## Figures and Tables

**Table 1:** Integration of radiological (RECIST 1.1) and pathological (EORTC) responses in nine adult STS patients enrolled in the UNRESARC phase II trial (NCT03651375). F1 = fusion-positive; F0 = fusion-negative; RVT = residual viable tumour; PR = partial response; SD = stable disease; PD = progressive disease.

Patient	Subtype	Grade	Fusion Status	Detected Fusion	Age/Sex	EORTC Grade (RVT%)	RECIST 1.1	Primary Site
P1	MPNST	G2	F0	None	68/F	C (9%)	PR	Forearm
P2	LMS	Ungraded	F0	None	65/M	D (30%)	PD	Thigh
P3	UPS	G3	F0	None	73/F	E (90%)	SD	Forearm
P4	MFS	G2	F0	None	68/M	E (70%)	PR	Arm
P5	UPS	G2	F0	None	54/M	D (35%)	SD	Arm
P6	UPS	G3	F1	JAZF1-MYH7B; PRKCA-associated	64/M	D (20%)	SD	Upper back
P7	MPNST	G3	F1	LINC01133-OGA	70/F	C (5%)	SD	Thorax
P8	MPNST	G3	F0	None	44/M	E (70%)	SD	Leg
P9	MFS	G3	F1	SGSH-PRKCA	66/M	D (10%)	SD	Thigh

**Table 2:** Comparison of guidelines regarding NGS use in non-specialised institutions and specialised reference centres [26,33]. NGS implementation is characterised by stratification: non-specialised institutions perform DNA-based hotspot screening for established ESCAT Level I/II targets, whereas reference centres are essential for RNA-based comprehensive profiling in complex-karyotype STS. This approach is known for detecting novel structural variants and enabling functional interpretation, bridging molecular discovery with precision therapy.

NGS	Non-Specialised Unit	Sarcoma Reference Centre
Indication	-Reserved for diagnostically ambiguous cases -Metastatic screening where actionable alterations may change systemic therapy	-Routine for all high-grade, complex-karyotypeSTS -Mandatory for clinical trial enrolment, novel targets, or RWE
Methods	-Targeted DNA panel (50–100 genes); hotspot mutations and copy-number alterations -Limited or no fusion detection	-RNA-based NGS (e.g., FusionPlex Sarcoma v2) + comprehensive DNA panel (300+ genes) -Captures fusions, mutations, indels, copy-number changes, MSI, TMB
ReportedAlterations	-ESCAT Level I/II: *ALK* (IMT), *COL1A1-PDGFB* (DFSP), *NTRK* fusions -MSI-H/dMMR, TMB-H	-Canonical + novel fusions; kinase fusions(*NTRK*, *ALK*, *ROS1*, *PRKCA*) -*TP53*, *IDH1/2*, *ATRX*, *CDKN2A/B* -Full ESCAT-tiered annotation with trial-matching
ClinicalUtility	-Guides standard therapy (e.g., imatinib for DFSP) -Complex cases referred to the sarcoma centre	-Precision oncology: biomarker-matched/off-label therapy, fusion inhibitors -Molecular tumour board, phase I/II trials, RWE for rare STS
Reporting	-7–10 days; standard report; optionalreview	-5–7 days; mandatory MDT -MDT interpretation; therapeuticrecommendations + trial options

Abbreviations: STS = soft tissue sarcoma; NGS = next-generation sequencing; IMT = inflammatory myofibroblastic tumour; DFSP = dermatofibrosarcoma protuberans; MSI-H = microsatellite instability-high; dMMR = mismatch repair deficient; TMB = tumour mutational burden; ESCAT = ESMO Scale for Clinical Actionability of molecular Targets; RWE = real-world evidence.

**Table 3:** Summary of GFs identified alongside the role of specific partners and probable implications [15,36,37,38,39,40,41,42]. (A) SGSH-PRKCA in MFS (P9) retaining sulfatase, type I phosphodiesterase, PRKCA C1, and kinase domains. (B) LINC01133-OGA in MPNST (P7), a lncRNA-enzyme fusion. (C) Concurrent JAZF1-MYH7B (chr7:27995037-chr20:33563203, out-of-frame but preserving myosin SH3/motor/tail domains) and PRKCA-associated intergenic rearrangements (chr1, retaining C1/kinase domains) in UPS (P6).

Fusion	Histology	5’ Partner (Function)	3’ Partner (Function)	Predicted Consequence	Biological Interpretation	Clinical Implications
*SGSH-PRKCA*	MFS G3	SGSH -lysosomal enzyme; heparan sulphate degradation	PRKCA - DAG/Ca^2+^-regulated serine-threonine kinase	In-frame status unknown; partial PRKCA C1/kinase domain retention	Possible PRKCA deregulation or truncation; gain/loss uncertain due to PKC autoregulation complexity - requires phosphorylation assays/localisation studies	Non-actionable; validate at protein p- .level (e.g., PKCα IHC/Western blot)
*LINC01133-OGA*	MPNST G3	LINC01133 - lncRNA; epigenetic modulator	OGA - O-GlcNAc hydrolase	Likely non-coding transcript; translation improbable	Transcriptional dysregulation of OGA locus over chimeric protein; may perturb O-GlcNAc cycling	No therapeutics; hypothesis-generating for metabolic/epigenetic profiling (e.g., GlcNAc-MS)
*JAZF1-MYH7B*	UPS G3	JAZF1 - PRC2-interacting transcriptional repressor	MYH7B - slow myosin heavy chain locus	Out-of-frame; truncated non-functional transcript	Probable passenger event; no preserved domains or sarcoma precedents	Clinically irrelevant; confirm via RNA-seq/protein (no preserved ORFs expected)
*PRKCA-intergenic*	UPS G3	Intergenic (chr1: AL137078.2/AL049541.1)	PRKCA	Truncation with kinase domain retention (low coverage)	Aberrant PRKCA transcription or rearrangement; impact unclear sans copy-number context	Non-actionable; orthogonal validation essential (qRT-PCR, FISH for CNV/PRKCA status)

## Data Availability

The case-level clinicopathological dataset and processed fusion-call summary underlying this article are available from the corresponding author on reasonable request, subject to institutional and ethical restrictions. Raw sequencing files are not publicly deposited because the consent obtained for this study does not permit open-access release of potentially identifiable genomic data.

## Abbreviations

The following abbreviations are used in this manuscript:

AI	anthracycline-ifosfamide
AMP	anchored multiplex PCR
CNA	copy number alteration
CNV	copy number variant
CTOS	Connective Tissue Oncology Society
DAG	diacylglycerol
DFSP	dermatofibrosarcoma protuberans
dMMR	mismatch repair deficient
E	EORTC no effect (residual viable tumour > 90%)
ECOG	Eastern Cooperative Oncology Group
ESCAT	ESMO Scale for Clinical Actionability of molecular Targets
EORTC	European Organisation for Research and Treatment of Cancer
FFPE	formalin-fixed paraffin-embedded
F0	fusion-negative
F1	fusion-positive
FISH	fluorescence in situ hybridisation
GF	gene fusion
gLOH	genome-wide loss of heterozygosity
GIST	gastrointestinal stromal tumour
HRD	homologous recombination deficiency
IMT	inflammatory myofibroblastic tumour
LMS	leiomyosarcoma
lncRNA	long non-coding RNA
MDT	multidisciplinary team
MENA	Middle East and North Africa
MFS	myxofibrosarcoma
MPH	molecular pathology histogram
MPNST	malignant peripheral nerve sheath tumour
MSI-H	microsatellite instability-high
NGS	next-generation sequencing
OS	overall survival
PD	progressive disease
PFS	progression-free survival
PR	partial response
PRC2	polycomb repressive complex 2
qRT-PCR	quantitative reverse transcription polymerase chain reaction
RWE	real-world evidence
RT	radiotherapy
RT-PCR	reverse transcription polymerase chain reaction
SD	stable disease
SNV	single-nucleotide variant
STS	soft tissue sarcoma
TMB	tumour mutational burden
UPS	undifferentiated pleomorphic sarcoma
VMAT	volumetric modulated arc therapy

## References

[1] Mavroeidis L., Napolitano A., Huang P., Jones R.L. (2024). Novel Therapeutics in Soft Tissue Sarcoma. Cancers.

[2] Choy E. (2018). Soft Tissue and Bone Sarcoma.

[3] Sbaraglia M., Bellan E., Dei Tos A.P. (2021). The 2020 WHO Classification of Soft Tissue Tumours: News and Perspectives. Pathologica.

[4] Remiszewski P., Filipek K., Pisklak A., Chmiel P., Rutkowski P., Czarnecka A.M. (2025). Neoadjuvant or Adjuvant Chemotherapy in Soft-Tissue Sarcoma?. Curr. Oncol. Rep..

[5] Dudzisz-Śledź M., Rogala P. (2019). Advances in Systemic Treatment of Advanced Soft Tissue Sarcomas. Oncol. Clin. Pract..

[6] Dancsok A.R., Asleh-Aburaya K., Nielsen T.O. (2017). Advances in Sarcoma Diagnostics and Treatment. Oncotarget.

[7] Stacchiotti S., Maria Frezza A., Demetri G.D., Blay J.-Y., Bajpai J., Baldi G.G., Baldini E.H., Benjamin R.S., Bonvalot S., Bovée J.V.M.G. (2022). Retrospective Observational Studies in Ultra-Rare Sarcomas: A Consensus Paper from the Connective Tissue Oncology Society (CTOS) Community of Experts on the Minimum Requirements for the Evaluation of Activity of Systemic Treatments. Cancer Treat. Rev..

[8] Trent J.C., Rosenberg A.E., Pollock R., Pollock R.E., Delaney T.F. (2020). Sarcomas: Evidence-Based Diagnosis and Management.

[9] Remiszewski P., Falkowski S., Szumera-Ciećkiewicz A., Spałek M.J., Rutkowski P., Czarnecka A.M. (2025). From Pathogenesis to the Patient’s Bedside: A Comprehensive Review of Extraskeletal Myxoid Chondrosarcoma. J. Cancer Res. Clin. Oncol..

[10] Dey M., Remiszewski P., Piątkowski J., Golik P., Teterycz P., Czarnecka A.M. (2025). MicroRNA Bioinformatics in Precision Oncology: An Integrated Pipeline from NGS to AI-Based Target Discovery. J. Appl. Genet..

[11] Diab T., Tarhini A., Jaber G., Raffoul C., Zeineddine N., Kreidieh L., Hemade A., Barake M., Saghieh S., Mahfouz R. (2026). Real-World Implementation of Next-Generation Sequencing in Sarcoma: Molecular Insights and Therapeutic Outcomes. Med. Sci..

[12] Pollock R.E., Randall R.L., O’Sullivan B. (2019). Sarcoma Oncology: A Multidisciplinary Approach.

[13] Warmke L.M., Folpe A.L. (2026). Fusion-Positive Soft Tissue Tumors: A Selective Review. Semin. Diagn. Pathol..

[14] Gündoğdu Y., Şenocak Taşçı E., Özer L., Boynukara C., Çeçen R., Mutlu A.U., Yıldız İ (2025). Next-Generation Sequencing-Based Genomic Profiling of Advanced Soft Tissue and Bone Sarcomas. Front. Oncol..

[15] Miettinen M., Felisiak-Golabek A., Luiña Contreras A., Glod J., Kaplan R.N., Killian J.K., Lasota J. (2019). New Fusion Sarcomas: Histopathology and Clinical Significance of Selected Entities. Hum. Pathol..

[16] Remiszewski P., Siedlecki E., Wełniak-Kamińska M., Mikula M., Czarnecka A. (2026). Choosing the Right Animal Model for Sarcoma Research. Cell. Mol. Life Sci..

[17] Casolino R., Beer P.A., Chakravarty D., Davis M.B., Malapelle U., Mazzarella L., Normanno N., Pauli C., Subbiah V., Turnbull C. (2024). Interpreting and Integrating Genomic Tests Results in Clinical Cancer Care: Overview and Practical Guidance. CA Cancer J. Clin..

[18] Gounder M.M., Agaram N.P., Trabucco S.E., Robinson V., Ferraro R.A., Millis S.Z., Krishnan A., Lee J., Attia S., Abida W. (2022). Clinical Genomic Profiling in the Management of Patients with Soft Tissue and Bone Sarcoma. Nat. Commun..

[19] Szurian K., Kashofer K., Liegl-Atzwanger B. (2017). Role of Next-Generation Sequencing as a Diagnostic Tool for the Evaluation of Bone and Soft-Tissue Tumors. Pathobiology.

[20] Spałek M.J., Koseła-Paterczyk H., Borkowska A., Wągrodzki M., Szumera-Ciećkiewicz A., Czarnecka A.M., Castaneda-Wysocka P., Kalinowska I., Poleszczuk J., Dąbrowska-Szewczyk E. (2021). Combined Preoperative Hypofractionated Radiotherapy With Doxorubicin-Ifosfamide Chemotherapy in Marginally Resectable Soft Tissue Sarcomas: Results of a Phase 2 Clinical Trial. Int. J. Radiat. Oncol. Biol. Phys..

[21] Eisenhauer E.A., Therasse P., Bogaerts J., Schwartz L.H., Sargent D., Ford R., Dancey J., Arbuck S., Gwyther S., Mooney M. (2009). New Response Evaluation Criteria in Solid Tumours: Revised RECIST Guideline (version 1.1). Eur. J. Cancer.

[22] Donato Di Paola E., Nielsen O.S., EORTC Soft Tissue and Bone Sarcoma Group (2002). The EORTC Soft Tissue and Bone Sarcoma Group. European Organisation for Research and Treatment of Cancer. Eur. J. Cancer.

[23] Serban B., Cursaru A., Iordache S., Cretu B., Nica M., Iacobescu G., Popa M., Radu E., Cirnu M., Cirstoiu C. (2026). Molecular Characterization of Soft Tissue Sarcomas Using RNA-Based Next-Generation Sequencing. Int. J. Mol. Sci..

[24] Sreenath N.D., Singh K., Rastogi S., Biswal N., Singh A., Barwad A., Mridha A.R., Gamanagatti S., Shamim S.A., Dharmashaktu Y. (2026). Clinical Impact of Integrating RNA-Based Next-Generation Sequencing Into the Diagnostic Evaluation of Soft Tissue Sarcomas: Insights From a Single-Center Multidisciplinary Workflow. JCO Glob. Oncol..

[25] Remiszewski P., Taczała J., Rosiński M., Szumera-Ciećkiewcz A., Szostakowski B., Czarnecka A.M. (2025). Dermatofibrosarcoma Protuberans (DFSP): Diagnostics and Molecular Pathology. Curr. Treat. Options Oncol..

[26] Mosele M.F., Westphalen C.B., Stenzinger A., Barlesi F., Bayle A., Bièche I., Bonastre J., Castro E., Dienstmann R., Krämer A. (2024). Recommendations for the Use of next-Generation Sequencing (NGS) for Patients with Advanced Cancer in 2024: A Report from the ESMO Precision Medicine Working Group. Ann. Oncol..

[27] Remiszewski P., Gaik W., Skora A., Wąż J., Filipek K., Pisklak A., Dudzisz-Śledź M., Rutkowski P., Czarnecka A. (2025). Selinexor in the Treatment of Liposarcoma: From Preclinical Evidence to Clinical Practice. Med. Oncol..

[28] Ottaiano A., Sabbatino F., Picone C., Di Carluccio N., Simonetti I., Di Mauro A., Tafuto S. (2026). Emerging Genomic and Immunological Correlates Defining Oligometastatic Trajectories in Intermediate/High-Grade Soft-Tissue Sarcomas. Genes.

[29] Ladanyi M., Antonescu C.R., Leung D.H., Woodruff J.M., Kawai A., Healey J.H., Brennan M.F., Bridge J.A., Neff J.R., Barr F.G. (2002). Impact of SYT-SSX Fusion Type on the Clinical Behavior of Synovial Sarcoma: A Multi-Institutional Retrospective Study of 243 Patients. Cancer Res..

[30] Bobak K., Tysarowski A., Seliga K.A., Pia̧tkowski J., Golik P., Spałek M.J., Szumera-Ciećkiewicz A., Rutkowski P., Czarnecka A.M. (2025). Identification of a Novel *PRUNE2::NTRK2* Gene Fusion in Soft Tissue Sarcoma Patients-Friend or Foe? Case Series. Ther. Adv. Med. Oncol..

[31] Zhang Q., Yang Y., You X., Ju Y., Zhang Q., Sun T., Liu W. (2023). Comprehensive Genomic Analysis of Primary Bone Sarcomas Reveals Different Genetic Patterns Compared with Soft Tissue Sarcomas. Front. Oncol..

[32] Kulaç İ., Bulutay P., Meriçöz Ç.A. (2021). What Would Next Generation Sequencing Bring to the Diagnosis and Treatment of Sarcomas? A Series of 20 Cases, a Single Institution’s Experience. Turk. Patoloji Derg..

[33] van de Haar J., Roepman P., Andre F., Balmaña J., Castro E., Chakravarty D., Curigliano G., Czarnecka A.M., Dienstmann R., Horak P. (2024). ESMO Recommendations on Clinical Reporting of Genomic Test Results for Solid Cancers. Ann. Oncol..

[34] Remiszewski P., Tysarowski A., Seliga K.A., Bobak K., Piątkowski J., Golik P., Spałek M.J., Szumera-Ciećkiewicz A., Wągrodzki M., Rutkowski P. (2025). Clinicopathological and Genomic Profiling in Undifferentiated Pleomorphic Sarcoma: Small Series, Clear Message. J. Appl. Genet..

[35] Kawano T., Inokuchi J., Eto M., Murata M., Kang J.-H. (2022). Protein Kinase C (PKC) Isozymes as Diagnostic and Prognostic Biomarkers and Therapeutic Targets for Cancer. Cancers.

[36] Chen N., Zhang Q., Sun L., You X., Chen S., Chen D., Yang F. (2025). Comprehensive Study of Gene Fusions in Sarcomas. Investig. New Drugs.

[37] Van A.-A.N., Kunkel M.T., Baffi T.R., Lordén G., Antal C.E., Banerjee S., Newton A.C. (2021). Protein Kinase C Fusion Proteins Are Paradoxically Loss of Function in Cancer. J. Biol. Chem..

[38] Hirose T., Ikegami M., Kojima S., Yoshida A., Endo M., Shimada E., Kanahori M., Oyama R., Matsumoto Y., Nakashima Y. (2023). Extensive Analysis of 59 Sarcoma-Related Fusion Genes Identified Pazopanib as a Potential Inhibitor to *COL1A1-PDGFB* Fusion Gene. Cancer Sci..

[39] Zhu G., Benayed R., Ho C., Mullaney K., Sukhadia P., Rios K., Berry R., Rubin B.P., Nafa K., Wang L. (2019). Diagnosis of Known Sarcoma Fusions and Novel Fusion Partners by Targeted RNA Sequencing with Identification of a Recurrent *ACTB-FOSB* Fusion in Pseudomyogenic Hemangioendothelioma. Mod. Pathol..

[40] Koontz J.I., Soreng A.L., Nucci M., Kuo F.C., Pauwels P., van Den Berghe H., Dal Cin P., Fletcher J.A., Sklar J. (2001). Frequent Fusion of the *JAZF1* and *JJAZ1* Genes in Endometrial Stromal Tumors. Proc. Natl. Acad. Sci. USA.

[41] Broadwell L.J., Smallegan M.J., Rigby K.M., Navarro-Arriola J.S., Montgomery R.L., Rinn J.L., Leinwand L.A. (2021). Myosin 7b Is a Regulatory Long Noncoding RNA (lncMYH7b) in the Human Heart. J. Biol. Chem..

[42] Zhang Z., Tan E.P., VandenHull N.J., Peterson K.R., Slawson C. (2014). O-GlcNAcase Expression Is Sensitive to Changes in O-GlcNAc Homeostasis. Front. Endocrinol..

